# Comparative analysis of pancreatic amylase activity in laboratory rodents

**DOI:** 10.1038/s41598-023-44532-6

**Published:** 2023-10-12

**Authors:** Linda F. Böswald, Ellen Kienzle, Dana Matzek, Marion Schmitz, Bastian A. Popper

**Affiliations:** 1 Chair for Animal Nutrition and Dietetics, Department of Veterinary Sciences, Faculty of Veterinary Medicine, Ludwig-Maximilians-Universität München, Munich, Germany; 2 Core Facility Animal Models, Biomedical Center, Faculty of Medicine, Ludwig-Maximilians-Universität München, Munich, Germany

**Keywords:** Physiology, Metabolism

## Abstract

Alpha-amylase is the main enzyme for starch digestion in the mammalian gastrointestinal tract. There are species differences in the enzymatic activity of pancreatic amylase that are related to the digestive strategy and natural diet of a species. This aspect is well investigated in pet and farm animals, while in common laboratory animal rodents, information is scarce. In the context of the 3R concept, detailed knowledge of the digestive physiology should be the basis of adequate nutrition, experimental planning and data interpretation. The present study aimed to obtain reference data on amylase activity in pancreatic tissue and duodenal digesta in laboratory mice, rats and hamsters. In addition, digesta was stained with Lugol’s iodine to visualize starch in the process of degradation throughout the gastrointestinal tract. Amylase activity in pancreatic tissue and duodenal digesta was significantly lower in hamsters than rats and mice. The Lugol staining showed intense starch degradation in the hamsters’ forestomachs, presumably by microbial fermentation. A possible explanation is that the prae-duodenal microbial starch fermentation enhances digestibility and reduces the need for pancreatic amylase in hamsters. Rats and mice may rely more on pancreatic amylase for prae-caecal starch digestion, while the microbial fermentation is mainly located in the caecum. The results clearly show species differences in the digestive capacity for starch in mice, rats and hamsters that need to be considered in the feeding of these species in the laboratory setting as well as in the use of rodents as translational animal models.

## Introduction

Alpha-amylase is the enzyme for prae-caecal digestion of starch in the gastrointestinal tract, which is predominantly secreted from pancreatic tissue in a biochemically active form induced by food intake. The enzyme cleaves the α-(1,4)-glycosidic bonds in the starch polysaccharide^1^. There are species differences in the sites of amylase synthesis, secretion and the levels of activity of this enzyme^1^.

Humans pancreatic amylase activity was reported to be on average 400 U/g pancreatic tissue in healthy subjects^2^. Human saliva also contains amylase, which begins to cleave starch during the chewing process^3^. The begin of starch degradation in the oral cavity contributes to the taste as well^4^.

The activity of pancreatic amylase of some animal species has been investigated under different dietary conditions and during maturation. In the omnivorous pigs, for example, the amylase activity was found to increase dramatically with age. In several studies, pancreatic amylase activity in pigs ranged between 1500 and 180,000 U/g pancreatic tissue^5–7^. Variations in amylase activity could be related to dietary starch content^8–10^ as major influencing factor. In the carni-omnivorous dogs, the activity of pancreatic amylase increases during growth and reaches its maximum of about 4600 U/g at 2 years of age^11^. Due to genetic changes during domestication of dogs^12,13^, amylase activity is surprisingly high and increasing with dietary carbohydrates, in contrast to the consistently low levels (~ 75 U/g) in the strictly carnivorous cats^11,14^. Dogs and cats do not possess salivary amylase^4^, while some herbivores and omnivores are known to secrete salivary amylase in various levels^1,15^. As herbivorous hindgut-fermenters, horses do not naturally ingest high starch diets. They have relatively low pancreatic amylase activities of approximately 80–900 U/g pancreatic tissue, which did not seem to be influenced by diet^16^.

Laboratory animals are commonly used as model organisms for human physiological and/or pathological states. Thus, knowledge about their physiologic processes is important to understand the functionality of the gastrointestinal tract. On the one hand, this is the prerequisite for adequate nutrition of the species in terms of animal welfare and the 3R concept^17^; on the other hand, detailed knowledge of the physiology of a laboratory animal species is essential to choose a suitable species for an experiment in any research area^18^, but especially so in any field that touches metabolism. The latter point contributes to the refinement of experimental planning and data interpretation, especially in the translational context.

Rats are one of the commonly used laboratory animal species. In rats, a clear induction of pancreatic amylase by carbohydrates in the diet was shown in a trial with high carbohydrate versus high lipid diets and intravenous infusion of different nutrient solutions^19^. There is also evidence for the presence of salivary amylase from the parotid gland in rats^20,21^. Data on the activity of pancreatic amylase under “normal laboratory conditions”, i.e. the feeding of standard, cereal-based laboratory rodent diets without any experimental intervention, are lacking. For mice, there is information on the genetic expression of amylase^22–24^. Several studies have explored the changes in amylase secretion under experimental conditions in vivo and in vitro^25–29^. However, to the authors’ information, reference values for pancreatic amylase activity under maintenance conditions are lacking.

Hamsters are used to model human pancreatitis and pancreatic cancer, among other research^30,31^. Ohbo et al.^32^ investigated the stimulation of pancreatic amylase in hamsters on a cellular level. It is also known that hamsters have genetic information encoding two types of salivary amylase and pancreatic amylase, but that there are differences between e.g. Chinese and Syrian hamsters^33^. Like in mice, there is a lack of reference data on pancreatic amylase activity in hamsters under standard feeding conditions in the laboratory setting.

Therefore, the aim of the present study was to gather data on pancreatic amylase activity in three commonly used laboratory rodent species—mice, rats and hamsters. To complement the enzyme activity data, the process of starch digestion throughout the gastrointestinal tract was visualized by microscopic evaluation. The combination of amylase activity data and visualization of starch in the digesta allows to understand the digestion process.

## Materials and methods

### Animals

Ethical approval was obtained (reference no. 203-25-02-20 from the Ethical Committee of the Veterinary Faculty, Ludwig-Maximilians-Universität München). Housing of laboratory animals was in accordance with European and German animal welfare legislations (5.1-231 5682/LMU/BMC/CAM 2019-0007) under specified-pathogen-free (SPF) conditions in individually ventilated cages (mice: type II long; rats: GR1800 Double decker; hamsters: type IIIH; Tecniplast, Buggugiate, Italy) on aspen granules bedding material (LAS bedding PG3, Altromin Spezialfutter GmbH Co., Lage, Germany). The cages were equipped with nesing material (5 × 5 cm Nestlet, Datesand, UK), a red corner house (Tecniplast) and play tunnels in different sizes for the hamsters and rats (Datesand). Room temperature ranged from 20 to 22 °C, relative from 45 to 55%. The light cycle was adjusted to 12 h light:12 h dark period. Room air was exchanged 11 times per hour and filtered with HEPA-systems. Hygiene monitoring was performed every 3 months based on the recommendations of the FELASA-14 working group.

For the study, 11 mice (inbred strain C57Bl/6J, 8-weeks-old), 11 rats (inbred strain LEW/Crl, 9-weeks-old), and 24 hamsters (outbred stock RjHan: AURA, 11 weeks old) were used. Mice and rats were purchased from Charles River (Germany), hamsters from Janvier (France). All animals were clinically healthy and had not been in any experiments beforehand. For at least 2 weeks before sacrifice, they were fed the same commercial pelleted breeding diet for rats and mice that is used as standard diet for these species in the facility (analyzed nutrient content see table 1). Due to the timing of the experiment, a new batch of the same diet had to be used for the hamsters (called “batch 2” in this context). Both batches of the diet were consistent in their nutrient profile, including starch content and degree of starch gelatinization, so that an influence of batch on the results can be excluded. Feed and water was available ad libitum and the animals were not fasted before they were sacrificed. The mice were killed by cervical dislocation, the rats and hamsters were euthanized (intraperitoneal injection of pentobarbital sodium at a dose of 500–800 mg/kg body weight (Release 300 mg/mL, WDT, Garbsen, Germany; no premedication). The procedure was standardized for all animals of the three species, starting with the transfer into transport cages in the early morning, where several pellets of the abovementioned diet were available. The animals were sacrificed in the morning, so that the timeframe was as short as possible (approximately 8–11 a.m.).

**Table 1:** Analyzed nutrient content of the diet.

Nutrient	Diet batch 1 Used for mice and rats	Diet batch 2 Used for the hamsters
[% as fed]		
Dry matter	90.0	90.1
Crude protein	22.7	21.8
Crude fat	5.1	4.7
Crude fiber	4.6	4.3
Crude ash	3.9	3.9
Nitrogen-free extracts	53.7	56.3
Starch	37.6	38.1
[% of starch]		
Degree of starch gelatinization	34.5	32.0

### Amylase assay

The animals were dissected immediately after sacrifice. At first, the pancreas was removed completely and separated from fat and connective tissue. Content from the anterior part of the duodenum was sampled. Both the total pancreas tissue and duodenal digesta were homogenized in buffer solution (distilled water with 0.9% NaCl, 0.2% bovine serum albumin, 20 mM CaCl_2_ based on the protocol given by Phadebas for their test kit) with a dispersing instrument (ULTRA TURRAX®, IKA®-Werke GmbH & CO. KG, Staufen, Germany) for ~ 1 min at the maximum level of the disperser, until no large particles were visible. The resulting homogenate was used for analysis of amylase activity with the Phadebas© assay (Phadebas AB, Kristianstad, Sweden). The test tablets contain a starch polymer, which bears a blue dye. Alpha-amylase in the sample hydrolyses the starch and releases the blue marker. The intensity of the blue colour in the solution was measured with a spectral photometer (GENESYS 10S UV-VIS, Thermo Scientific) twofold and the mean value of extinction was used to translate into amylase activity (U/L) from the standard curve given in the test protocol.

### Statistics

Amylase activity in pancreatic tissue and duodenum content, respectively, was compared between species via one-way ANOVA on ranks (prism GraphPad 5.04., GraphPad Software, San Diego, CA, USA). This test was chosen because not all data was normally distributed (Shapiro–Wilk normality test). The significance level was defined as α = 0.05 for all statistical tests.

### Microscopy

Representative digesta samples from the following anatomical sites were obtained: stomach (in rats and hamsters forestomach and glandular stomach), duodenum, caecum, colon. The samples were stored frozen at − 20 °C until further investigation. A small portion of each thawed sample was stained with 1 mL Lugol’s iodine (solution of 0.5 g iodine, 1 g potassium iodide in 600 mL distilled water) to detect starch by blue staining. The stained digesta samples were examined macroscopically and then with the stereomicroscope to qualitatively detect starch and describe its characteristics in the respective samples (microscope: Zeiss Stemi 508, Carl Zeiss Microscopy GmbH, Göttingen; software: ZEN core version 3.4.94.00001, Carl Zeiss Microscopy GmbH).

### Ethical approval

Ethical approval for the use of animals was obtained (reference no. 203-25-02-20 from the Ethical Committee of the Veterinary Faculty, Ludwig-Maximilians-Universität München). Housing of laboratory animals was in accordance with European and German animal welfare legislations (5.1-231 5682/LMU/BMC/CAM 2019-0007). The study is in accordance with the ARRIVE guidelines.

## Results

### Amylase activity

**Figure 1:**
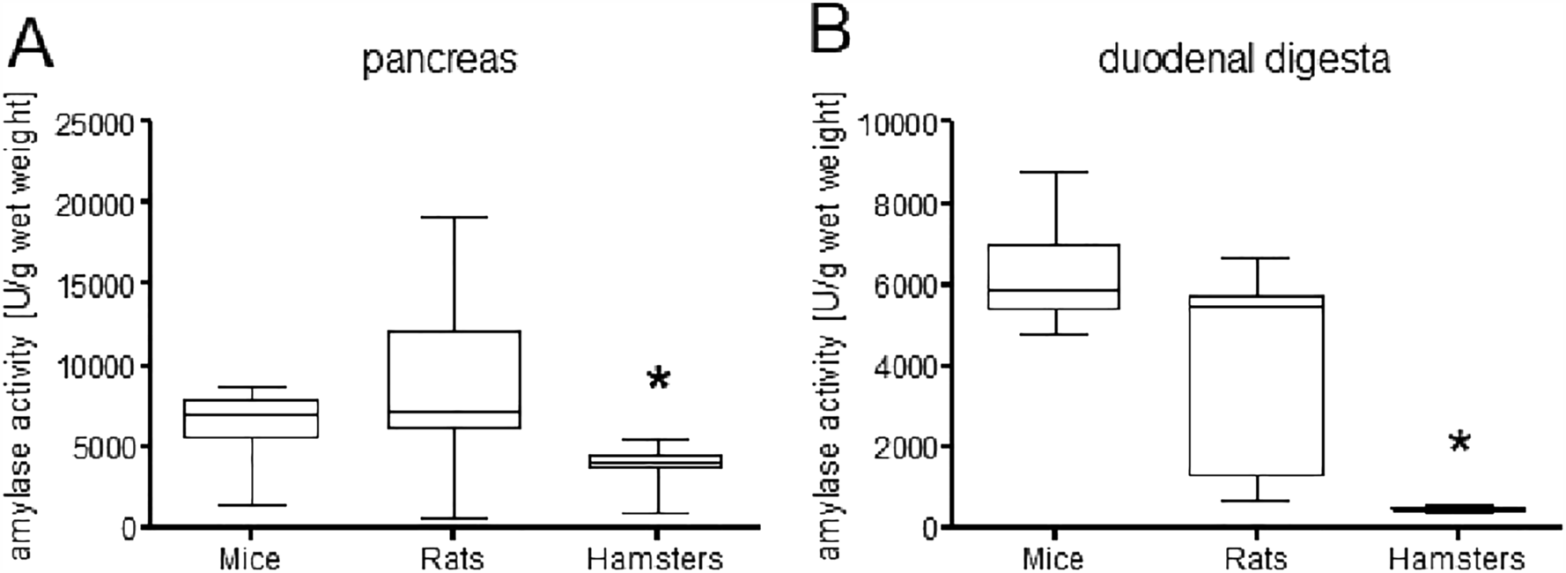
Comparison of pancreatic amylase activity (U/g wet weight) between the species in (**A**) pancreatic tissue and (**B**) duodenal digesta. In both samples, hamsters (n = 24) had significantly lower values than rats (n = 11) and mice (n = 11; *p* < 0.01).

Pancreatic amylase activity was significantly lower in hamsters (means ± SD: 3885 ± 964.5 U/g wet weight) than rats (9167 ± 5680 U/g wet weight) and mice (6281 ± 2493 U/g wet weight; *p* < 0.001; Figure 1A). Rats and mice did not differ significantly (*p* > 0.05). The rats had a broad range of amylase activity (662–19191 U/g wet weight), whereas in hamsters the variation was smallest (900–5423 U/g wet weight).

The same pattern was found in the duodenal digesta (hamsters: 450.8 ± 55.20 U/g wet weight vs. mice: 685 ± 1159 U/g wet weight and rats: 4027 ± 2367 U/g wet weight; *p* < 0.01; Figure 1B). Hamster values showed much lower variation then the other two species, with the broadest range in rats.

### Starch staining

**Figure 2:**
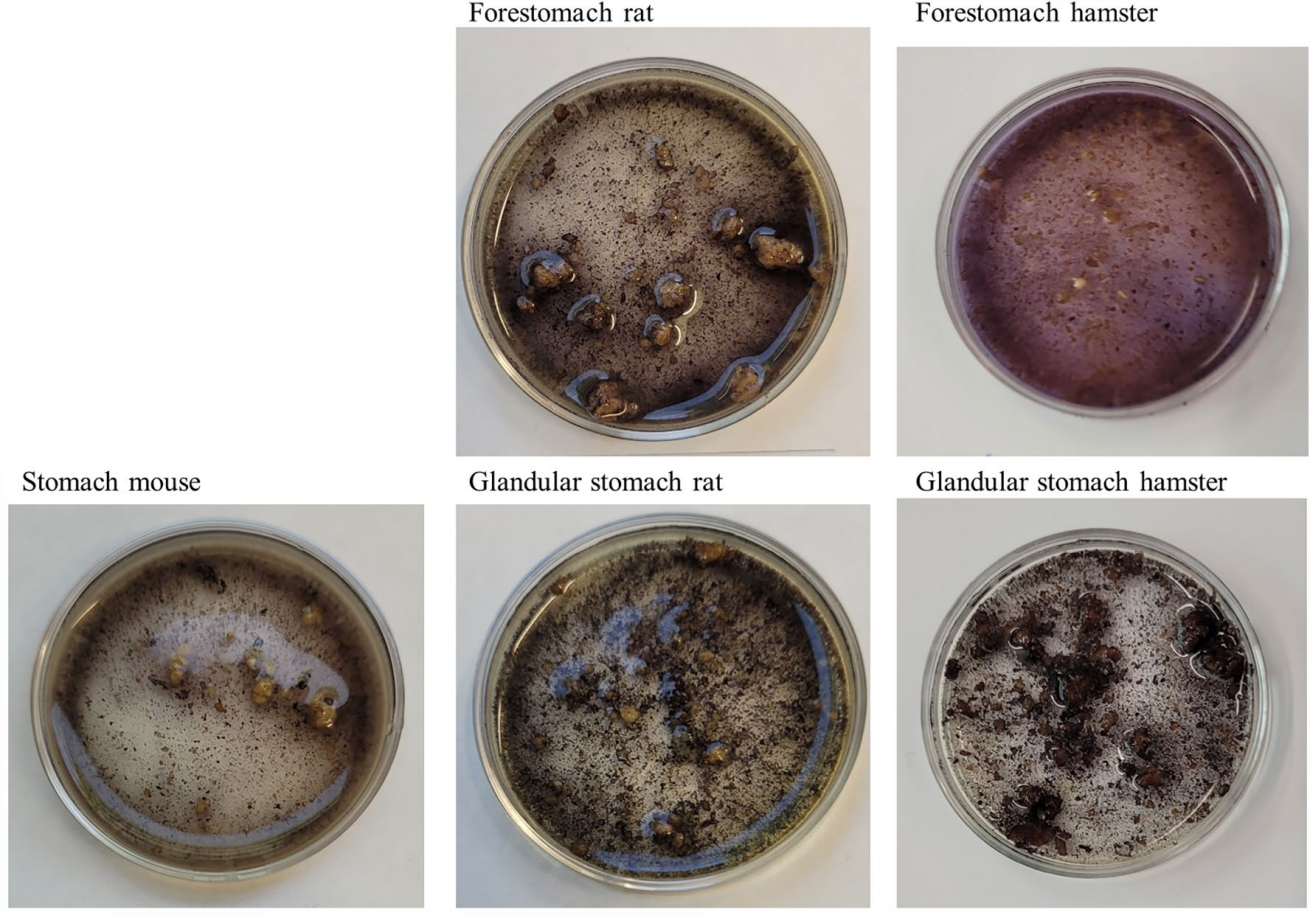
Macroscopic images of stained digesta of the stomachs in mice, rats and hamsters. Dark blue/violet staining indicates starch.

**Figure 3:**
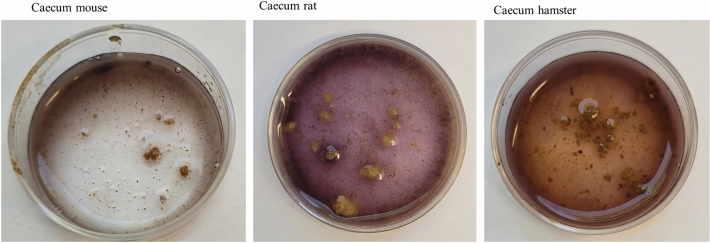
Macroscopic images of stained caecum content of mice, rats and hamsters. Blue/violet staining indicates starch.

**Macroscopic staining**. In mice, the stomach content showed dark blue stained starch particles and only slight staining of the fluid phase (Figure 2). In rats, where a separation of forestomach and glandular stomach content was possible, the forestomach content was stained dark blue with a slight blue tinge of the fluid phase, while in the glandular stomach content, only the particles were stained dark and the fluid phase was clear. The forestomach digesta of hamsters was of a “softer” and finer consistency than the more compact content of the glandular stomach. The hamsters’ forestomach digesta showed intense violet staining of the fluid phase and blue staining of particles. In the digesta from the glandular stomach of the hamsters, particles were stained dark blue, while the fluid phase was clear. In the small intestinal digesta of all three investigated species, there were not many stained particles visible and the fluid phase was not stained at all. The caecum content (Figure 3) was intensely stained with a violet fluid phase and dark particles, in all three species. In the colon content of mice, rats and hamsters, there was hardly any staining visible.

**Figure 4:**
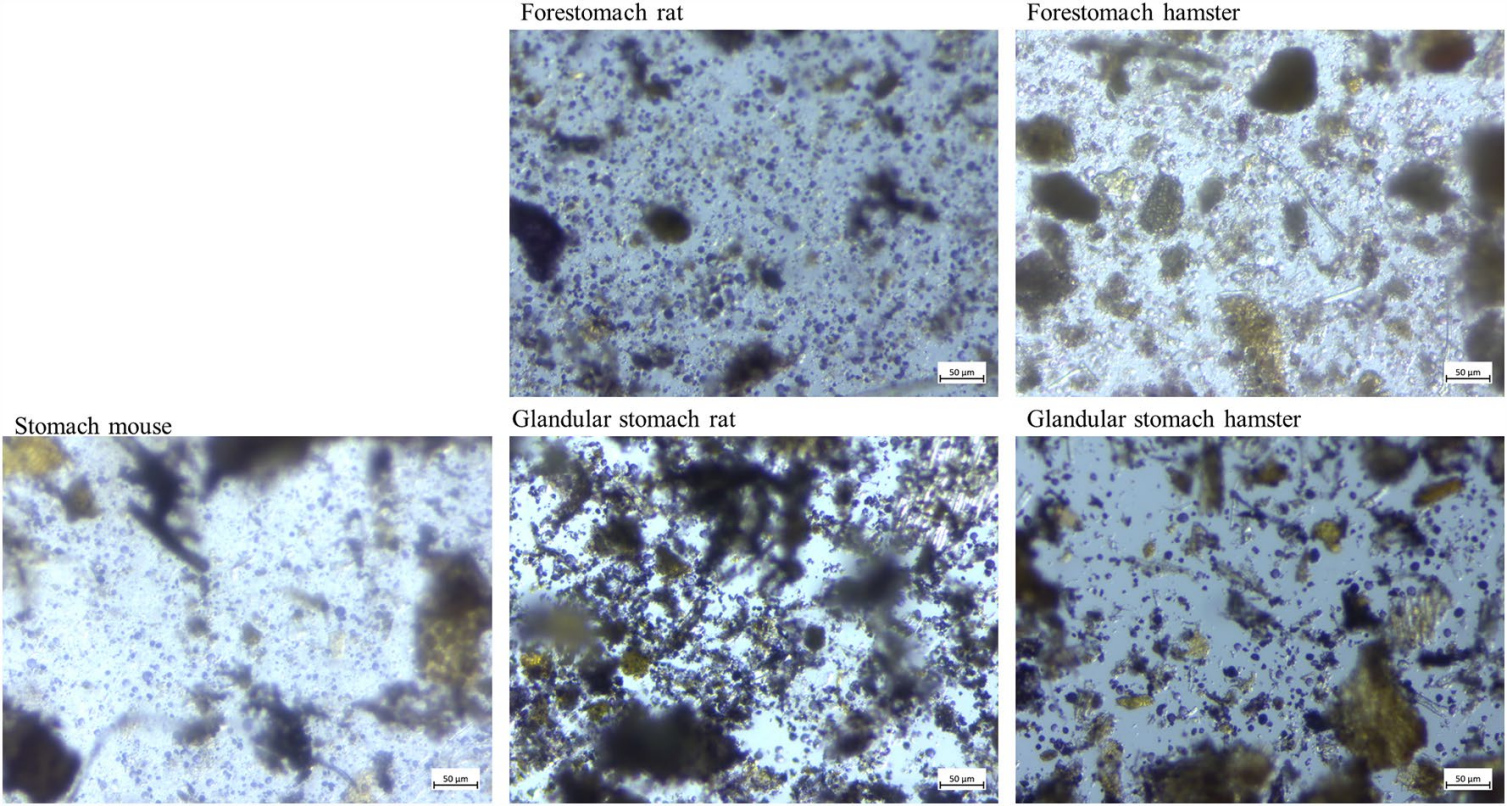
Stereomicroscopic images of stomach and forestomach content of mice, rats and hamsters. Starch is stained blue/violet with Lugol’s iodine.

**Figure 5:**
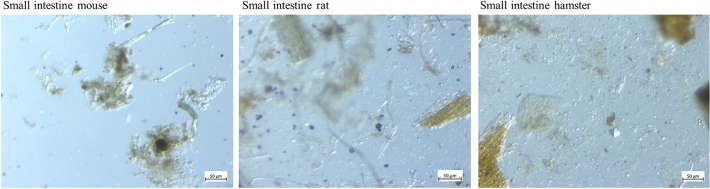
Stereomicroscopic images of small intestinal content of mice, rats and hamsters. Small, round starch particles are visible by blue stain.

**Stereomicroscopy**. The stomach samples of the mice showed a large amount of blue stained, round particles (Figure 4). In rats and hamsters, the glandular stomach samples were similar to the stomach samples of mice. The forestomach samples of rats and hamsters were hard to get into focus because of the tinge of the fluid phase. Stained starch particle sin various sizes were present. In the small intestinal content of mice, rats and hamsters, only a small number of round, stained particles was visible (Figure 5). The caecum content of all three species was hard to get into focus under the microscope, because the fluid phase was visible with a light violet stain. Violet particles of irregular shape were abundant in these samples. In the colon content samples of mice, rats and hamsters, only singular blue stained particles were visible.

## Discussion

Amylase activity in pancreatic tissue and duodenal digesta was analyzed in mice, rats and hamsters. In all three species, amylase activity was lower in the digesta samples than in the pancreatic tissue, which was to be expected due to dilution in the intestinal content. Due to the treatment of the diets before use in the SPF husbandry (pelleting, irradiation, gas atmosphere), enzyme activity from natural sources [e.g. 34] that may be found in the digesta under other circumstances may be negligible in laboratory animal husbandry. To minimize potential effects of diurnal variation in amylase secretion, all animals were sacrificed during the same time of day. The number of animals was the same in mice and rats, but higher in hamsters. The variation of results was higher in rats than both other species, irrespective of the number of individuals, and lowest in hamster in spite of the higher number. Thus, the variation in number of individuals does not seem to be the major influence on the results.

Compared to other species (Table 2), all three rodent species had lower pancreatic amylase activity than pigs^5–9^. Rats and mice showed higher pancreatic amylase activity than horses^16^, cats^14^ and dogs^11^, which have all been determined with the same method^35^. Hamsters fall in the same range as observed in adult dogs fed carbohydrate-containing diets^11^.

**Table 2:** Overview of literature data on pancreatic amylase activity in different species.

Species	Method	Pancreatic amylase activity *[U/g wet weight]*	Reference
Cat (*Felis catus*)	Phadebas®	74 ± 42	^14^
Cattle (*Bos taurus*)	Teco diagnostics kit Dahlqvist method	71–217 388–620	^43^ ^44^
Horse (*Equus caballus*)	Phadebas®	277 ± 265	^16^
Human	Boehringer Mannheim test	418	^2^
Hamster (*Mesocricetus auratus*)*	Phadebas®	3885 ± 964	Present study
Dog (*Canis lupus)*	Phadebas®	4662 ± 781	^11^
Mouse (*Mus musculus*)*	Phadebas®	6281 ± 2492	Present study
Rat (*Rattus norvegicus*)*	Phadebas®	9167 ± 5680	Present study
Pig (*Sus scrofa*)	Phadebas® Amylochrome Iodine-marker	2076–182,000 22,220–29,648 ~ 1500–3700	^6^ ^7^ ^5^

*Line names: hamster: Rj:Han:AURA; mouse: C57Bl/6 J; rat: Lewis.

Salivary amylase activity was not measured in the present study. In mice, rats and hamsters, the presence of salivary amylase has been shown^1^. In a qualitative literature synopsis by Boehlke et al.^1^, and an overview by Ohya et al.^34^, hamsters and rats ranged similar, while the salivary amylase activity in mice seems to be a bit lower. However, the variability of analysis method has to be taken into consideration. Salivary amylase can contribute to the very first step of starch degradation. Due to the rather short time of the ingesta in the oral cavity, the part in the overall digestion is low. In hamster, it may be possible that the enzyme can degrade starch in the cheek pouches during storage or in the forestomach, in combination with microbial fermentation.

Rats, mice and hamsters seem to be adapted to a natural diet that contains grains as sources of native starch by relatively high pancreatic amylase activity in combination with microbial fermentation, partly in the non-glandular stomach and partly in the large intestine.

In both pancreatic tissue and duodenal digesta, hamsters had significantly lower enzyme activity than mice and rats, which did not differ significantly. There was also much less inter-individual variation in the hamster samples (Figure 1), compared to higher variation in the amylase activity measured in the samples of rat pancreatic homogenates and prae-caecal digesta. The stomach of hamsters is distinctly compartmentalized with a large forestomach and a glandular stomach portion of roughly the same size^36,37^. This differentiation is much less pronounced in rats and mice^37–39^. The hamster forestomach is a first site of microbial fermentation^36^, which becomes clear when looking at the digesta from both stomach parts. The digesta in the forestomach is finer and moister than the “harder” content of the glandular stomach part (own observation from dissections), which indicates different processes in the two stomach compartments. Lugol stain of the digesta from both parts of the hamster stomach (Figs. 2 and 4) showed an intense violet stain of the fluid phase in the forestomach content, indicating the solubility of starch, which is being degraded by microbial fermentation. When the structure of the compact starch granules is broken down and the cleavage of starch molecules begins, a kind of gelatinous smear in the fluid phase becomes visible. Through Lugol’s staining, this becomes visible in a light violet colouration and focusing the image via the stereomicroscope becomes harder. This was the case in the hamsters’ forestomach content.

In contrast, the digesta from the glandular part of the hamster stomach did not show signs of degradation or solubility (Figs. 2 and 4). An explanation for the low pancreatic amylase activity in hamsters as compared to rats and mice may be the high capacity for microbial starch fermentation in the forestomach^36^. If the carbohydrate fraction of the diet is already in the process of digestion in the anterior part of the gastrointestinal tract, it may reach the site of absorption in the small intestine without the need for high pancreatic enzyme activity. The starch staining supports this by indicating the presence of less starch in the large intestine. In rats and mice, on the other hand, the capacity for fermentation in the non-glandular portion of their stomach seems to be limited, thus increasing the need of pancreatic amylase activity to enable starch digestion. The non-glandular region is muss less compartmentalized than in the hamster and a separation into the two stomach regions is not given in the same way. Thus, the passage through this region is faster and the capacity for microbial fermentation more limited. Prae-caecally undigested starch will reach the hindgut with the caecum as major site of microbial fermentation^40–42^. It can be expected that these differences in the site of aut-enzymatic digestion (by the organism’s own enzymes) and microbial fermentation lead to species differences in the intermediate metabolism. If in hamsters, starch is microbially degraded to a larger extent than in rats and mice, a lower duodenal glucose absorption would be expected. Rats and mice, on the other hand, will rely more on the microbial hindgut fermentation. Given the highly digestible, semi-purified nature of the lab diet fed in this experiment, all animals seemed to have adapted to the high starch content well and without obvious digestive problems. It cannot be excluded, however, that other types of diets are not suitable for the three rodent species and that species-specific diets should be considered.

## Conclusions

The present study confirms species differences in pancreatic and duodenal amylase activity between three commonly used laboratory rodent species, rats, mice and hamsters. A potential explanation for the lower values in hamsters is the difference in stomach morphology and functionality. Hamsters have a pronounced forestomach as site of microbial fermentation, which may reduce the need for pancreatic amylase. These findings need to be considered in choosing species-specific diets and when using rodents in animal experiments, especially in the field of metabolism. In the context of the 3R principles, this contributes to the *Refinement* of laboratory animal feeding and experimental planning.

## Data Availability

The original data is available from the authors upon reasonable request (linda.boeswald@lmu.de).
